# Diagnostic difficulties of *Lactobacillus casei *bacteraemia in immunocompetent patients: A case report

**DOI:** 10.1186/1752-1947-2-315

**Published:** 2008-09-30

**Authors:** Chiara Tommasi, Francesco Equitani, Marcello Masala, Milva Ballardini, Marco Favaro, Marcello Meledandri, Carla Fontana, Pasquale Narciso, Emanuele Nicastri

**Affiliations:** 1 National Institute for Infectious Diseases, INMI 'L. Spallanzani', via portuense, 00149 Rome, Italy; 2 S. Filippo Neri General Hospital – Department of Transfusion Medicine and Hemotherapy, via Martinotti, 00135 Rome, Italy; 3 S. Filippo Neri General Hospital, via Martinotti, 00135 Rome, Italy; 4 Department of Microbiology, Polyclinic of 'Tor Vergata', viale Oxford, 00133 Rome, Italy

## Abstract

**Introduction:**

Lactobacilli are currently proposed as probiotic agents in several dietary products. In blood cultures, they are usually considered as contaminants, but in recent years they have been recognized as causal infectious agents of endocarditis, urinary tract infections, meningitis, intra-abdominal infections and bacteraemia.

**Case presentation:**

We report a case of *Lactobacillus casei *bacteraemia in a 66-year-old immunocompetent man with a history of fever of unknown origin. Leuconostoc bacteraemia was demonstrated by blood culture, but a later polymerase chain reaction analysis with sequencing of 16S ribosomal RNA identified *Lactobacillus casei *and a successful antibiotic therapy was performed.

**Conclusion:**

Bacteraemia caused by probiotic organisms is rare but underestimated, since they are normally regarded as contaminants and their role as primary invaders is not always easily established. Although the consumption of probiotic products cannot be considered a risk factor in the development of diseases caused by usually non-pathogenic bacteria, specific individual clinical histories should be taken into account. This report should alert both clinicians and microbiologists to the possibility of unusual pathogens causing serious illnesses and to the use of 16S ribosomal RNA sequencing for molecular identification as a powerful tool in confirming the diagnosis of infrequent pathogens.

## Introduction

Bacteraemia due to lactobacilli is uncommon among immunocompetent patients. One of the current limitations in the laboratory processing of blood cultures is the requirement to subculture isolates in order to perform further testing necessary for bacterial identification. This often results in one or more days of delay in the aetiological diagnosis and errors in identification can occur [1].

Lactobacilli are ubiquitous in the environment. In humans, they colonize the oral cavity, gastrointestinal tract, and vagina. In general, lactobacilli have little or no virulence, but have been recognized as opportunistic pathogens in some reports [2-9]. The clinical significance of *Lactobacillus *bacteraemia in immunocompetent patients is not clear, but considering the wide distribution of *Lactobacillus *and the relatively few infections they cause, these bacteria can be considered to have very little virulence in healthy humans.

## Case presentation

A 66-year-old man was referred to the Haematological and Transfusional Medicine Department of an urban general hospital in December for persistent fever and night sweating. The patient was affected by hypertension, diverticulosis, haemorrhoidal bleeding and by chronic obstructive pulmonary disease with periodic exacerbations.

Starting in June, the patient reported weakness, increased blood glucose serum levels and decreased body weight. In November, swelling of superficial lymph nodes, fever and nocturnal sweating had occurred. The laboratory test results showed normocytic normochromic anaemia (haemoglobin, Hb, 9.5 g/dl), white blood cell count (WBC) was 7170/mm^3 ^with 85% neutrophils and 11% lymphocytes, 7.13 mg/dl C-reactive protein (CRP) with normal values < 0.8 mg/dl, erythrocyte sedimentation rate (ESR) of 59 mm/hour, fibrinogen 597 mg/dl (normal range 150–450) and beta 2-microglobulin 5.21 mg/litre (normal values 1.42–3.21 mg/litre). Levels of Ca 15.3, Ca 19.9, PSA, TPA, alpha-fetoprotein, FT3, FT4, TSH, vitamin B12 and folic acid were in the normal range. Serological tests for CMV, *Toxoplasma gondii*, VZV, Brucella, HBV, HCV and HIV were negative. The Tuberculin Sensitivity Test with 5 IU of Purified Protein Derivative (PPD) was performed with negative results. Markers for autoimmune diseases (ANA, APCA, ASMA, and ENA) were in the normal range.

In December, because of persistent fever, weakness, nocturnal sweating and loss of 7 kg in body weight, the patient was referred to the Haematological Department. Blood examinations documented a persistent increase in ESR (90 mm/hour) and CRP (6.7 mg/dl) levels and a progressive haemoglobin decrease (nadir 8 g/dl); dosage of cytokines showed increased levels of IL-6 (12.7 pg/ml, normal values < 4.1), IL-10 (20.5 pg/ml, normal values < 9.1) and TNF-alpha (32.4 pg/ml, normal values < 8.1); the study of B- and T-lymphocyte subpopulations by RT-PCR (CD5, CD19, CD20, Sig, CD3, CD7, TCR) gave normal results. Blood transfusions and iron therapy were performed. In January, total body computed tomography (CT) scanning showed cardio- and splenomegaly. Non-complicated diverticulosis in the descending colon and sigmoid colon and anal haemorrhoids were revealed by colonoscopy. A trans-oesophageal echocardiogram showed mitral prolapse with regurgitation but no valvular vegetation was found. A dental X-ray was negative for dental abscess or caries.

In February, the patient was referred to the Infectious Disease Department. On admission, four consecutive blood cultures were performed. Gram-positive aerobic-anaerobic coccobacillary bacteria were isolated from all specimens and identified as *Leuconostoc *spp. resistant to vancomycin and trimethoprim-sulphamethoxazole. Therapy with parenteral levofloxacin (500 mg intravenous bid) was started. After 2 weeks of parenteral therapy, the patient's conditions improved, but because of persistence of fever and increased levels of ESR (59 mm/hour), the therapy was continued with oral moxifloxacin. A blood culture specimen was examined with PCR for sequencing of 16S ribosomal RNA. The molecular analysis did not confirm the previous diagnosis and the microorganism was identified as *Lactobacillus casei*.

In March, haemorrhoidectomy was performed. During the postoperative period, the patient developed respiratory failure with an increased ESR (100 mm/hour). Chest CT scanning revealed multifocal pneumonia. Blood and sputum culture results were negative. A 7-day course of parenteral therapy with amoxicillin-clavulanate (2 g bid) was added to the moxifloxacin. In April, after 6 weeks of fluoroquinolone therapy, the patient was feeling well: fever, weakness and nocturnal sweating had resolved, the haemoglobin and CRP serum levels were in the normal range, ESR was reduced (51 mm/hour) and antimicrobial therapy was stopped.

Three months after the end of antibiotic therapy, the patient was hospitalized because of a new episode of multifocal pneumonia, likely to be secondary to a previous upper respiratory tract infection. During the hospitalization, an abdomen CT scan demonstrated normalization of spleen size. A trans-thoracic echocardiogram confirmed the presence of cardiomegaly and mitral prolapse with regurgitation. Blood and sputum cultures were negative. The patient was successfully treated with intravenous ampicillin-sulbactam (2/1 g tid) and oral levofloxacin (500 mg qd). No microorganisms were isolated in blood, sputum or urine.

Six months after the bloodstream infection with *Lactobacillus casei*, the patient reported a marked increase in body weight with complete normalization of ESR (8 mm/hour), CRP (0.5 mg/dl), and Hb values (13 g/dl). Now the patient is on the waiting list for cardiac valvular replacement.

## Discussion

Bacteraemia results in significant morbidity and mortality, especially among immunocompromised patients, but it is uncommon among immunocompetent people without risk factors for bloodstream infections [1].

Lactobacilli are Gram-positive, catalase-negative, non-sporulating, aerobic or facultative anaerobic, rod-shaped bacteria. They are ubiquitous and inhabit a wide variety of habitats, including the gastrointestinal tract, oral cavity and vagina. Moreover, these bacteria are traditionally used in the manufacture of fermented foods and as probiotic [2].

Among pathogenic species in humans, *Lactobacillus casei *appears to be the most frequently isolated, although *L. paracasei *and *L. rhamnosus *are also encountered in clinical situations [2]. However, isolates are often only identified by genus and the most automated identification systems are not capable of accurate differentiation of *Lactobacillus *species. These microorganisms are rarely infectious and their presence as commensals in the gastrointestinal tract is associated with protection against pathogens, stimulation of the immune system and positive effects on colonic health and host nutrition.

Although lactobacilli are usually considered contaminants in blood cultures, they have been identified in some clinical reports as causal agents of dental caries, infectious endocarditis, urinary tract infections, chorioamnionitis, endometritis, meningitis, intra-abdominal, liver or spleen abscesses, and bacteraemia [2-7]. Commonly, these infections can be correlated with previous illnesses (recent surgery, transplants, valvulopathy, diabetes mellitus, AIDS and cancer) or with either immunosuppressive therapy or antibiotic treatment, which could promote the development or the selection of the microorganism.

*Leuconostoc *are Gram-positive, non-motile, aerobic-anaerobic facultative, coccobacillary bacteria members of the Streptococcaceae family, environmental organisms commonly found in vegetable matter, in milk products and other chilled food products. These pathogens can often be mistaken by automated identification systems that are not capable of accurate differentiation.

In our report, a temporal correlation was reported between consumption of probiotic lactobacilli and bacteraemia in the presence of intestinal disease. Because of the previous diagnosis of diverticulosis, the patient had frequently eaten probiotic dietary products with *Lactobacillus casei *before the onset of fever. Recently, reports have increased of *Lactobacillus *infections after probiotic lactobacilli intake, particularly in the paediatric populations with underlying diseases, for example in short-gut syndrome [8,9]. In our patient, underlying diseases such as endocarditis, abscess, neoplasia or chronic bowel disease were excluded. Nevertheless, in some reports, diverticulosis was reported as the cause of bacteraemia in immunocompetent people [10]. In our patient, it is possible that intestinal diverticulosis, with local mucositis, could represent the intestinal entry site of the *Lactobacillus casei *in the bloodstream. The determination of faecal calprotectin, a biomarker of altered intestinal permeability state, has not been performed and without data on the calprotectin level, no major consideration can be raised on the role played by increased permeability states [11].

Bacteraemia caused by probiotic organisms is rare but underestimated, since they are normally regarded as contaminants and their role as primary invaders is not always easily established. Although the increased consumption of dairy products does not appear to promote an increase in *Lactobacillus *sp. bacteraemia, it is noteworthy that the probiotic strain of *Lactobacillus rhamnosus *has been involved in several bacteraemia cases and *Lactobacillus *populations can survive in the gastrointestinal tract of humans after oral administration [8].

The identification of unusual disease agents by conventional methods involves some degree of subjective evaluation and the conventional procedures used in most Italian hospitals are not specifically designed for it. As the 16S ribosomal RNA nucleotide sequence is available in databases for most bacterial species, 16S ribosomal RNA sequencing becomes a valuable identification strategy for such microorganisms [12].

## Conclusion

This report of a case of *Lactobacillus casei *bacteraemia in an immunocompetent individual should alert both clinicians and microbiologists to the possibility of unusual pathogens causing serious illnesses; also, 16S ribosomal RNA molecular identification seems to represent a powerful tool in the confirmation of diagnosis of infrequent pathogens.

## Competing interests

The authors declare that they have no competing interests.

## Authors' contributions

CT, FE, MM, PN and EN participated in the case study, followed the clinical improvement of the patient and helped with drafting of the manuscript. MB, MF, MM and CF carried out the microbiological studies. All authors read and approved the final manuscript.

## Consent

Written informed consent was obtained from the patient for publication of this case report. A copy of the written consent is available for review by the Editor-in-Chief of this journal.
